# The Case for Improved Diagnostic Tools to Control Ebola Virus Disease in West Africa and How to Get There

**DOI:** 10.1371/journal.pntd.0003734

**Published:** 2015-06-11

**Authors:** Arlene C. Chua, Jane Cunningham, Francis Moussy, Mark D. Perkins, Pierre Formenty

**Affiliations:** 1 Medécins Sans Frontières, Access Campaign, Geneva, Switzerland; 2 Department of Infectious Diseases, Institute of Infectious Diseases and Epidemiology, Singapore; 3 Global Malaria Programme, World Health Organization, Geneva, Switzerland; 4 Department of Essential Medicines and Health Products, World Health Organization, Geneva, Switzerland; 5 Foundation for Innovative New Diagnostics, Geneva, Switzerland; 6 Department of Pandemic and Epidemic Diseases, World Health Organization, Geneva, Switzerland; Tulane School of Public Health and Tropical Medicine, UNITED STATES

The Ebola virus disease (EVD) outbreak in West Africa, with the widest and most intense transmission occurring in Guinea, Liberia, and Sierra Leone, has claimed more than 8,000 lives since it began in December 2013. It has had a massive impact on already fragile health systems and now threatens food security. The latest World Health Organization (WHO) report has shown that there has been a decrease in the cases in January 2015 [1].

Since the identification of Ebola in Guinea in March 2013, rapid deployment of international mobile laboratories through WHO networks—Global Outbreak Alert and Response Network (GOARN) [2] and Emerging and Dangerous Pathogens Laboratory Network (EDPLN) [3]—has been vital to outbreak control operations. Deployable laboratories from multiple international organizations have been established near Ebola treatment centers (ETC) in Guinea, Liberia, and Sierra Leone. The organizations providing laboratories are China Centers for Disease Control Lab, European Union Mobile Laboratory Consortium (EM Lab), Institute Pasteur Dakar, Institute Pasteur Lyon, Institute Pasteur Paris, Institut National de Recherche Bio-Médicale Mobile lab in Democratic Republic of Congo, National Institute for Communicable Diseases in South Africa, Public Health England Mobile lab, Public Health Canada Mobile Lab, Russian Rospotrebnadzor Mobile Lab, United States Centers for Disease Control (CDC), US First Area Medical Laboratory, US National Institutes of Health, and US Naval Medical Research Center Mobile Lab.

The primary function of these laboratories has been to confirm disease in patients with suggestive symptoms in order to trigger isolation and contact tracing and to document cure and/or non-infectiousness in survivors. Current diagnostic testing is performed exclusively using reverse transcription polymerase chain reaction (RT-PCR) on RNA extracted from venous blood samples collected by personnel wearing full personal protective equipment. In the absence of simpler methods, diagnosis of EVD in this outbreak has, until now, relied exclusively on testing conducted in these internationally run, mobile laboratories.

However, several technical and social factors conspire to delay diagnosis, starting with weak surveillance systems and slow patient access to centralized ETCs. While the mean processing time is 5 hours (time difference from when samples are received in the laboratory to when they are tested), there is a marked difference in the time from when the samples are collected from suspected patients to the time they are received by the laboratory. That time difference varies between 1 to 32 days, with a mean of 1.5, 1.8, and 2.1 days for Guinea, Liberia, and Sierra Leone, respectively. (unpublished, Pierre Formenty, WHO). There is a need to ameliorate transport of samples to the laboratories. Without the possibility of rapid, local confirmed diagnosis, patients are often reluctant to go to testing sites because of fear of contracting the disease during involuntary delays. Partly because of these delays, by the time of diagnosis the majority of patients with confirmed EVD have been symptomatic (and infectious) for 5–6 days [4]. Recent analysis by WHO of data collected from March 2014 to January 2015 from the three most affected countries found that the time difference between the date of onset of symptoms and the date of sample collection from suspected patients varies between 1 and 21 days, with a mean of 7.2 days. (Unpublished, Pierre Formenty, WHO). The lack of proven interventions (treatment and vaccines) makes early identification and isolated management of EVD cases with rapid and accurate diagnostics even more fundamental to interrupting disease transmission and bringing the outbreak under control [5]. Early detection and care is critical for transmission interruption, initiation of contact tracing, and accurate epidemiological surveillance and has been associated with improved prognosis.

What strategies can lead to safe and faster diagnosis? Though, clearly, cases are clustered—as of October 14, 90% of cases were reported from 14 districts [6]—new foci of transmission constantly emerge, and the changing distribution of cases suggests that it will be unwise to rely on a limited number of high-throughput laboratories and that more decentralized testing could decrease delays in notification. Delays described above are serious, as multiple studies have shown that in the first 2 days of symptomatology, some patients may have very high loads of circulating virus and thus are prone to spread EVD in the family and community [4,7]. The current centralized diagnostic testing strategy is adequate for case confirmation and proof of cure. But given the EVD geographic spread, the loss of many chains of transmission and the high number of unidentified cases, the current strategy might not be adequate for providing the kind of rapid and flexible response that would help stop Ebola transmission.

Certainly, we should continue with current surveillance strategy of active identification of cases and contact tracing. But in addition, we should establish in non-Ebola health care facilities a complementary surveillance system using safely administered point-of-care diagnostic tests to screen patients for non-Ebola infections and to accelerate detection of hidden and unknown chains of Ebola transmission.

Indeed, the majority of fevers and other non-specific symptoms seen in the outbreak area are not caused by EVD and may be due to one of various other infections endemic in the region, including malaria, typhoid fever, shigellosis, cholera, leptospirosis, rickettsiosis, relapsing fever, meningitis, hepatitis, and other viral hemorrhagic fevers. Among patients with EVD, co-infections with malaria are not uncommon (internal Medécins Sans Frontières report: Epi Bulletin Ebola Epidemic in West Africa). Having a point-of-care test could facilitate the safe reopening of non-Ebola health facilities that have been closed because of the outbreak. In the last quarter of 2014 in Liberia, 62% of health facilities were closed. In the three countries heavily affected by EVD, there has been a significant drop in outpatient visits, institutional deliveries and childhood immunizations [8]. It is important that health care workers dealing with patients outside ETCs have the capacity to exclude EVD without being exposed to additional risks of acquiring EVD.

More than 800 health care workers have reportedly been infected with EVD, and initial analysis shows that a substantial proportion of infections occurred outside the context of Ebola treatment and care centers [1,9]. Although the exact proportion is unknown, the frequency of reports of health care workers becoming infected through their work in routine medical practice outside ETCs is a telling commentary on the need to couple strengthening of infection prevention practices and extension of Ebola diagnostic testing more broadly into district or local health centers. Rapid, field-adapted point-of-contact/care tests that are highly predictive of EVD and do not carry extensive biosafety requirements could drastically improve detection of EVD patients through decentralized diagnostic testing.

Unfortunately, there is currently no commercially available test with stringent regulatory approval that will meet the needs of most decentralized testing centers. However, this is primarily a financial and logistical problem, not a technical one. For other diseases such as HIV, tuberculosis, and malaria, diagnostic assays with adequate performance based on detection of antigens or nucleic acid sequences have been developed and successfully introduced, with proper shepherding from the public sector [10–12]. A number of semi-portable molecular amplification tests platforms that integrate sample processing and greatly simplify PCR procedures have been designed and developed in recent years, including for diseases prevalent in resource-limited settings. Such devices can potentially be used for EVD detection, if adapted to the appropriate biosafety requirements. Alternatively, antigen detection, which has worked relatively well—albeit in a cumbersome ELISA format, could form the basis for a rapid diagnostic test (RDT) that, if sensitive and specific enough, could be valuable. The type of sample to be used (e.g., blood versus saliva) and the sample collection requirements (finger prick versus phlebotomy) will be key features for the tests to be simple enough for use in disseminated, low-resource locations.

In recognition of the urgent need for improved Ebola diagnostics, and to guide diagnostic research and development (R&D), a consensus target product profile (TPP) has been developed by the authors and other key partners (Table 1). The intended use of the profiled product is to distinguish symptomatic patients with acute EVD infection from those with non-Ebola virus infection. The TPP outlines two sets of test characteristics: desired (ideal) and acceptable, both of which would allow for varying degrees of decentralized EVD testing through reduced requirements for laboratory infrastructure and for technical expertise in sample collection and/or running the assay. However, in both cases, the requirement for very high test sensitivity and specificity is maintained, due to the serious individual and public health impacts of both false-positive and false-negative EVD results.

**Table 1:** Target product profile for *Zaire ebolavirus*: rapid, simple test to be used in the control of the Ebola outbreak in West Africa.

Key Features	Desired	Acceptable
**Priority Features**		
**Target population**	Patients presenting with fever to health care facilities for assessment.	
**Target use setting**	Decentralized health care facilities with no laboratoryinfrastructure available	Decentralized health care facilities with minimum laboratory infrastructure available
**Intended use**	In Ebola outbreak setting, distinguish between symptomatic patients with acute Ebola virus infection and non-Ebola virus infection without the need for confirmatory testing	In Ebola outbreak setting, distinguish between symptomatic patients with acute Ebola virus infection and non-Ebola virus infection with the need for confirmatory testing
**Clinical sensitivity** ^**a**^ ^**,**^ ^**b**^	>98%	>95%
**Analytical specificity**	>99%	>99%
**Type of analysis**	Qualitative or Quantitative	Qualitative
**Sample type**	Capillary whole blood from finger stick once/if the use of this type of samples has been validated	Whole blood from phlebotomy, in particular if collection is simple and automated to reduce biosafety requirements
	Other, less invasive sample types (e.g., saliva, buccal) once/if their use has also been validated	
**TEST PROCEDURE**		
**Number of steps to be performed by operator**	<3	<10
(use of different reagents/incubation steps)	0 timed steps	1 timed step
**Biosafety** ^**c**^	No additional biosafety in addition to Personal Protective Equipment^**c**^	No additional biosafety in addition to Personal Protective Equipment^**c**^
**Need for operator to transfer a precise volume of sample**	No	Acceptable if adequate disposable blood transfer device is provided
**Time to result**	<30 minutes	<3 hours
**Internal control**	included	included
**Sample preparation**	None or fully integrated	None or fully integrated
Need to process sample prior to performing the test		
**Operational Characteristics**		
**Operating conditions**	5–50°C	5–40°C
	90% RH	90% RH
**Reagent storage (stability)**	24 months at 40°C + 90% RH; no cold chain should be required	12 months at 30°C + 70% RH including 3 months at 40°C, no cold chain should be required
	Should be able to tolerate stress during transport (3 days at 50°C)	Should be able to tolerate stress during transport (3 days at 50°C)
**In-use stability (under tropical conditions)**	>1 hour for single use test after opening the pouch	>½ hour for single use test after opening the pouch
**Reagents reconstitution**	All reagents ready to use	Reconstitution acceptable if very simple to do.
Need to prepare the reagents prior utilization		All liquids, including water, already in kit
**Training needs**	Less than half a day for any level health care worker. Job aid provided	Less than 2 days for any level of health care worker. Job aid provided
Time dedicated to training session for end users		
**Equipment (if needed)**	Small and portable, handheld instrument	Small, table-top device, portable
	Weight <2 kg	
**Power requirements**	None required	110–220 V AC current
	Optional: 110–220 V AC current	DC power with rechargeable battery lasting up to 8 hours of testing
	DC power with rechargeable battery lasting up to 8 hours of testing	
**Need for maintenance/spare parts**	None	1 annual calibration ideally by operator

^a^ Clinical sensitivity in first 10 days of presentation. Allow for repeat testing as per WHO guidelines.

^b^ Reference test: Lab-validated quantitative PCR assay on blood sample (whole blood or plasma) drawn by phlebotomy.

^c^ Biosafety resources for Ebola: http://www.who.int/csr/disease/ebola/en/; http://www.who.int/csr/resources/publications/ebola/filovirus_infection_control/en/

Concurrently, WHO has also launched an emergency assessment, quality assurance mechanism for EVD diagnostics to inform procurement.

Rapidly developing and providing access to diagnostic tests aligned with the proposed TPP will require that research and development move at an accelerated pace. Continued coordination from WHO will be needed in order to achieve this goal, including such actions as:

- Developing consensus target product profiles to inform diagnostic developers about the specific needs
- Creating and distributing RNA and/or protein standards for early R&D support and analytical testing
- Creating and coordinating a virtual specimen bank network, to ensure equitable access to clinical and virological materials for assay development and evaluation
- Establishing ethical and pragmatic standards for rapid assay endorsement
- Conducting laboratory studies to assess analytical performance
- Conducting field studies to demonstrate clinical performance and feasibility of implementation in Ebola settings
- Deploying teams to support training of local health worker teams to safely use the tests in the early implementation phase

There are no good technological reasons why rapid diagnostics for use in decentralized locations cannot be developed to help stop this outbreak. To achieve this goal, the field needs a rapid mobilization of resources and coordination of work to accelerate the development and validation of these urgently needed tools, as well as equitable access. To help achieve this, WHO is providing this coordinating role in partnership with Foundation for Innovative New Diagnostics (FIND), Médecins Sans Frontières (MSF), and the multiple actors involved with the EVD response.
